# First isolation and molecular confirmation of *Brucella canis* in dogs from Egypt

**DOI:** 10.1186/s12866-026-04787-1

**Published:** 2026-02-20

**Authors:** Ahmed Thabet, Ahmed Orabi, Nour H. Abdel-Hamid, Lamia Ali El-Ebeedy, Mona Kadry, M. E. Hashad

**Affiliations:** 1 Department of Microbiology, Faculty of Veterinary Medicine, Cairo University, PO Box 12211, Giza, Egypt; 2 WOAH Reference Laboratory for Brucellosis, Department of Brucellosis Research, Agricultural Research Center, Animal Health Research Institute, P.O. Box 264‑, Giza, 12618 Egypt; 3 Department of Microbiology, Faculty of Pharmacy, New Valley University, El-Kharga, Egypt; 4 Department of Zoonoses, Faculty of Veterinary Medicine, Cairo University, PO Box 12211, Giza, Egypt

**Keywords:** Brucellosis, Dog, ERIC-PCR, Suis-ladder multiplex PCR, Egypt

## Abstract

**Background:**

Brucellosis, a disease of major veterinary and public health concern, remains widespread in several regions, including Egypt. This study reports the isolation and molecular confirmation of *Brucella canis* from dogs in Egypt and provides genetic relatedness of *Brucella melitensis* and *Brucella canis* in dogs to evaluate their public health significance.

**Methods:**

A total of 785 dogs (both stray and owned) in Cairo and Giza (Feb–Dec 2024) were examined for *Brucella spp.*; all blood samples were examined using serological tests only (RBPT, SAT with 2-ME) however, 365 reproductive tissue samples including 215 and 150 from testicular and uterine samples respectively were collected from animals undergoing surgical procedures and were used for bacteriological isolation. Molecular confirmation was achieved via AMOS-PCR and Suis-ladder multiplex PCR for *B. canis*. Genetic relatedness was assessed by ERIC-PCR fingerprinting and cluster analysis, with Simpson’s diversity index used to evaluate typing discrimination.

**Results:**

Twelve *Brucella spp.* isolates (3.28%) recovered, comprising 8 isolates for *B. melitensis* biovar 3 and 4 for *B. canis*. *B. melitensis* occurred in both stray and owned dogs while *B. canis* was found only in strays marking its first isolation in Egypt. Suis-ladder PCR reliably distinguished *B. canis* from *B. suis*, and ERIC-PCR showed moderate genetic diversity. Temporal–spatial data revealed low, but the detection occurred across several districts in both owned and stray dog populations.

**Conclusion:**

Molecular tools like Suis-ladder PCR and ERIC-PCR proved effective for precise characterization of isolates in addition, this study documents the first isolation of *B. canis* from dogs in Egypt emphasizing their role as potential public health relevance of canine brucellosis.

**Supplementary Information:**

The online version contains supplementary material available at 10.1186/s12866-026-04787-1.

## Background

Brucellosis is a zoonotic microbial disease that is caused by many *Brucella spp.* that affect multiple mammalian hosts. In recent decades, interest in *Brucella spp.* has grown due to their dual significance in both veterinary and public health fields. Globally, the prevalence of brucella infection varies widely, with reported seroprevalence in livestock ranging from less than 1% in some European countries to over 15% in parts of Africa and Asia [1].

Human brucellosis remains endemic in many Mediterranean, Middle Eastern, and African countries, where the close contact between humans and animals, combined with traditional livestock management practices, facilitates transmission [2].

In Egypt and other countries of the Middle East, *B. melitensis* in addition *B. abortus* are the greatest frequently stated types, posing a continuous challenge due to their zoonotic potential [3].

The detection and characterization of *Brucella spp.* have undergone significant advancements over time. Historically, diagnosis relied on conventional culture methods and biochemical identification, which, although considered the gold standard, were hindered by slow growth rates and the need for high-containment laboratory facilities due to the pathogen’s infectious nature [4]. Serological assays provided faster screening tools but often required confirmatory testing due to cross-reactivity [5].

More recently, molecular investigation has revolutionized *Brucella spp.* detection, enabling rapid, sensitive, and specific identification directly from clinical and environmental samples [6]. The integration of classical bacteriological methods with advanced molecular tools now provides a more inclusive consideration of the epidemiology of the pathogens, antimicrobial resistance and genetic diversity, which is crucial for control and eradication programs [7].

Among the *Brucella spp*, *B. canis* is a well-recognized cause of reproductive disorders in dogs, especially in kenneled populations. Infection in bitches may lead to abortion and stillbirth, whereas infected males often present with epididymitis, prostatitis, or orchitis [8, 9]. Bacteria can persist in the host even after antibiotic therapy, and shedding may occur via saliva, nasal secretions, urine, and reproductive fluids also, male dogs may remain infectious post-castration, as *B. canis* can keep on in the prostate and lymphoid tissues [10, 11].

Although *B. canis* is established as a pathogen in animals, its significance in human brucellosis is still unclear. Just a few mild cases in human have been described, and many are likely overlooked due to vague clinical manifestations and low clinical suspicion [12–14]. Infected individuals may present with undulating fever, chills, general weakness, splenomegaly, and enlarged peripheral lymph nodes. Confirmation of the disease still depends on bacterial isolation, which remains the diagnostic gold standard [15].

Brucellosis diagnosis in dogs is challenging. The organism is isolated from body secretions that remains the definitive method [17]. Serological screening tests are useful for early detection but lack sensitivity in chronic cases, and a highly sensitive and specific assay is still unavailable. However, bacteremia may be intermittent, leading to false negatives [16]. PCR-based detection offers a rapid and specific alternative, but its use is still largely confined to research settings [18–20]. Because data on *B. canis* in dogs are limited, this study investigated the occurrence, molecular features, and genetic relatedness of *B. melitensis* and *B. canis* in dogs in Egypt and to assess their public health implications.

## Materials and methods

### Samples collection and processing

A total of 785 dogs (both stray and owned) were examined during the study period; however, 365 reproductive tissue samples including 215 and 150 from testicular and uterine samples respectively were collected only from animals undergoing surgical procedures and were used for bacteriological isolation. Stray dogs were sampled at two shelters (involved in the capture–neuter–vaccinate–release program for street dogs), while owned dogs were sampled at private veterinary clinics in Cairo and Giza Metropolises. Samples were collected aseptically during surgical procedures. After overnight fasting, the dogs were anesthetized using a general anesthetic regimen. Premedication was performed with atropine sulfate (0.1 mg/kg body weight, subcutaneous injection; Atropine Sulphate, El Nasr Pharm. Chem. Co., Egypt). Xylazine (1 mg/kg, intramuscular injection; Xylaject, ADWIA Co., Egypt) was administered 15 min before induction. Anesthesia was induced and maintained with ketamine hydrochloride (10 mg/kg, intravenous injection, 10% solution; Ketamine Alfasan, Elyoser, Egypt) (Nesgash et al., 2016). During the procedures, physiological parameters including oxygen saturation (SpO₂), heart rate, respiratory rate, and body temperature were continuously monitored. Lactated Ringer’s solution was administered intravenously to maintain hydration, and postoperative analgesia was provided using meloxicam (0.2 mg/kg, subcutaneous injection). All anesthetic and surgical procedures were approved by the Institutional Animal Care and Use Committee (IACUC) in the Faculty of Veterinary Medicine, Cairo University in Egypt (Vet CU081020251256). Blood samples (from all examined dogs (*n* = 785)) were aseptically obtained from the cephalic vein of each animal for subsequent serological testing only. Sampling was conducted over an eleven-month period, from February to December 2024. All specimens were transported under cold chain conditions to the Brucellosis Department, Animal Health Research Institute (Dokki, Giza), for serological examination and bacterial isolation in a biosafety level 3 (BSL-3) laboratory.

### Serological examination

Testing of Serum samples for brucella antibodies were performed by using the Rose Bengal Plate Test (RBPT) with rough antigen, as previously described [21, 22]. In addition, the Serum Agglutination Test (SAT) with 2-mercaptoethanol (2-ME) was performed following the protocol of *Alton et al.* [23]. Serum titers < 50 were considered negative, titers ≥ 50 and < 200 were considered inconclusive, and titers ≥ 200 were interpreted as positive [24]. The *B. canis* rough antigen was prepared from the reference strain RM666 (National Veterinary Services Laboratory, Ames, IA, USA). Briefly, bacterial cells were suspended in formalinized phosphate-buffered saline (PBS) and heat-killed as described by *Hamdy et al.* [25].

### Bacterial isolation and phenotypic identification

Blood samples collected from all examined dogs (*n* = 785) were used for serological testing only and were not cultured for brucella isolation. Reproductive tissues (testicular and uterine samples) were processed for bacteriological isolation as described by the OIE (2021). Surfaces of testicular and uterine samples were sterilized by immersion in absolute ethanol followed by flaming, then aseptically dissected into small fragments and homogenized in sterile phosphate-buffered saline (PBS). A loopful of homogenate was streaked onto tryptic soy agar (TSA; Oxoid, UK) supplemented with selective antibiotics (polymyxin B, bacitracin, cycloheximide, nalidixic acid, nystatin, and vancomycin; Cat. No. SR0083). The Plates were then incubated at 37 °C for up to 14 days, with duplicate plates incubated in ambient air and in an atmosphere enriched with 10% CO_2_. Suspected brucella colonies were identified based on colony morphology and biochemical reactions, including oxidase, catalase, and urease tests. Colony smoothness or roughness was determined by acriflavine agglutination [26].

### Molecular identification of Brucella isolates

DNA was extracted through boiling method. The isolates were initially identified to the species level using AMOS-PCR [27]. The reaction mixture (25 µl) contained 1× PCR master mix (Genedirex, South Korea), 0.2 µM of each primer (Table 1), and 0.5 µl (10 ng) of DNA template. Amplification products were resolved on 1% agarose gels in 1× TAE buffer alongside a 100 bp DNA ladder (Genedirex, South Korea; Cat. No. DM101-0100).

**Table 1:** Suis-ladder PCR primers, targeted genes and expected amplicon sizes

Primer	5’-3’ nucleotide sequence	Target gene	Amplicon size in bp	Reference
BMEI1426	TCGTCGGTGGACTGGATGAC	Deletion of 351 in BMEI1426-BMEI1427 bp in *B. canis*	774	[28]
BMEI1427	ATGGTCCGCAAGGTGCTTTT			
BR1080f	CCCTTGGTTTGTAGCGGTTG	Deletion of 162 bp in BR1080 in *B. abortus* and *B. melitensis*	197	
BR1080r	TCATCGTCCTCCGTCATCCT			
BMEI1688	TCAATCGCGTGAACAATGCT	Deletion of 20,883 bp in BMEI1674-BMEI1703 in *B. suis* and *B. canis*	278	
BMEI1687	GCGGGCTCTATCTCAAGGTC			
BMEI0205f	CGTCAACTCGCTGGCCAAGAG	Derived from VNTR Bruce 11	551, 299, 425, 614	
BMEI0205r	GCAGGAGAACCGCAACCTAA			

### Molecular Confirmation of *B. canis* by Suis-Ladder Multiplex PCR

The Suis-ladder multiplex PCR assay was performed for confirmation of *B. canis* isolates, as the species produces a characteristic profile under the same amplification conditions used for *B. suis* [28]. PCR was carried out in a 25 µl mixture containing 3 µl of DNA template, 12.5 µl MyTaq™ HS Red Mix (Meridian Bioscience, USA), 1 µl (12.5 pmol) of each primer (Table 1), and 1.5 µl nuclease-free water. The thermal cycling program consisted of initial denaturation at 95 °C for 7 min, followed by 30 cycles of denaturation at 95 °C for 35 s, annealing at 63 °C for 45 s, and extension at 72 °C for 60 s, with a final extension at 72 °C for 6 min.

### ERIC-PCR and phylogenetic analysis

ERIC-PCR fingerprinting was performed on DNA from the recovered brucella strains and reference strains from livestock. Each 25 µl reaction contained 1× PCR master mix (Genedirex, South Korea), 0.4 µM of each primer (ERIC1 and ERIC2), and 10 ng DNA template, following [29]. PCR products were separated on 1% agarose gels in 1× TAE buffer and visualized using a gel documentation system. Banding patterns were analyzed with GelJ v.2.3 [30] using the Jaccard coefficient (1% tolerance) and UPGMA clustering. Simpson’s diversity index [31] was calculated to assess discriminatory power.

## Results

### Serological findings

All 785 serum samples (both stray and owned) tested by Rose Bengal Plate Test (RBPT) and Serum Agglutination Test (SAT) with 2-mercaptoethanol (2-ME) were seronegative for brucella antibodies. No agglutination reactions were observed in any of the examined samples, indicating the absence of detectable anti-brucella antibodies among both stray and owned dogs.

### Brucella isolation

In this study all confirmed bacterial isolates originated from reproductive tissues samples only as in Table 2where from a total of 365 reproductive samples (215 testicles and 150 uteri) collected from male and female dogs, 12 bacterial isolates (3.28%) were confirmed as *Brucella spp*. Colonies displayed typical morphology. All isolates were oxidase-, catalase-, and urease-positive, and *B. melitensis* isolates were smooth and reacted with anti-A and anti-M antisera, whereas *B. canis* isolates exhibited rough colony morphology and reacted with anti-R monospecific serum by acriflavine testing. Of the 12 isolates, 7 were recovered from testicular samples (2.74%) and 5 from uterine samples (3.33%). All isolates were capable of growth on media containing thionin and basic fuchsin dyes (1/50,000), did not need CO_2_ for growth, and were negative in hydrogen sulfide (H_2_S) production.

**Table 2:** Bacteriological and molecular identification of brucella isolates recover from stray and owned dogs

Colony morphology (Acriflavine test)	Specimen	Gender	Isolates’ No.	AMOS-PCR	BcSS-PCR	Urease test	Lysis by phages			CO2 requirement	Thionin	Basic Fuchsin	H_2_S	Monospecific antisera			Interpretation
							Iz	Tb	*R*/C					A	M	*R*	
**S**	Uterus	Female (Stray)	1	731 bp	-	+ (24 h)	+	-	-	-	+	+	-	+	+	-	*B. melitensis* Bv3
**S**	Testicles	Male (Stray)	2	731 bp	-	+ (24 h)	+	-	-	-	+	+	-	+	+	-	*B. melitensis* Bv3
**S**	Testicles	Male (Stray)	3	731 bp	-	+ (24 h)	+	-	-	-	+	+	-	+	+	-	*B. melitensis* Bv3
**S**	Testicles	Male (Owned)	4	731 bp	-	+ (24 h)	+	-	-	-	+	+	-	+	+	-	*B. melitensis* Bv3
**S**	Testicles	Male (Owned)	5	731 bp	-	+ (24 h)	+	-	-	-	+	+	-	+	+	-	*B. melitensis* Bv3
**S**	Uterus	Female (Stray)	6	731 bp	-	+ (24 h)	+	-	-	-	+	+	-	+	+	-	*B. melitensis* Bv3
**S**	Testicles	Male (Stray)	7	731 bp	-	+ (24 h)	+	-	-	-	+	+	-	+	+	-	*B. melitensis* Bv3
**S**	Uterus	Female (Stray)	8	731 bp	-	+ (24 h)	+	-	-	-	+	+	-	+	+	-	*B. melitensis* Bv3
**R**	Uterus	Female (Stray)	9	-	300 bp	+ (45 min)	-	-	+	-	+	-	-	-	-	+	*B. canis*
**R**	Testicles	Male (Stray)	10	-	300 bp	+ (45 min)	-	-	+	-	+	-	-	-	-	+	*B. canis*
**R**	Uterus	Female (Stray)	11	-	300 bp	+ (45 min)	-	-	+	-	+	-	-	-	-	+	*B. canis*
**R**	Testicles	Male (Stray)	12	-	300 bp	+ (45 min)	-	-	+	-	+	-	-	-	-	+	*B. canis*

### Molecular identification and biotyping

**Fig. 1:**
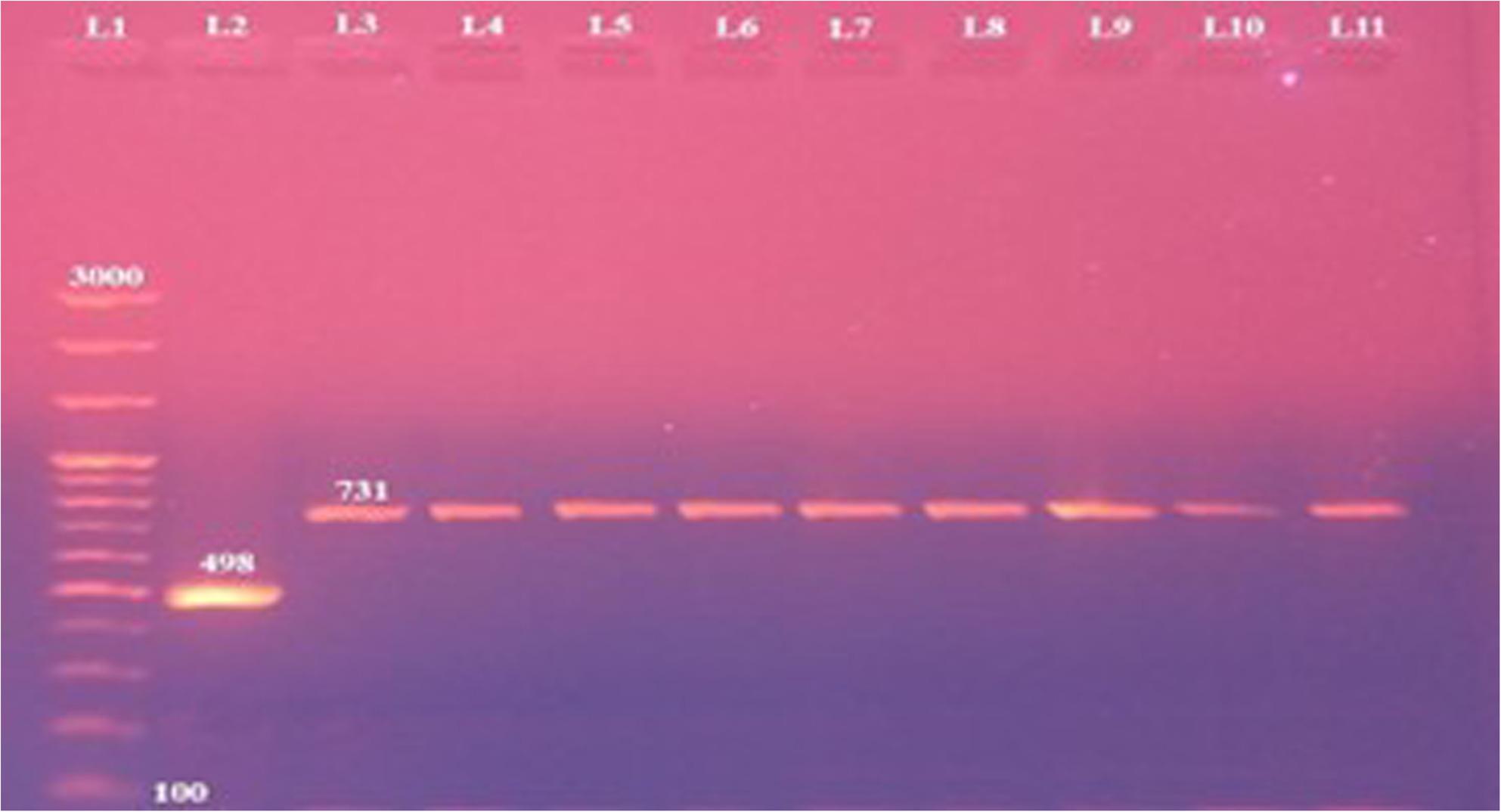
Differentiation of Brucella spp. by AMOS-PCR. Lane 1: 100 bp DNA size marker, lane 2: B. abortus reference strain 544; lane 3: B. melitensis reference strain Ether and lanes 4-11: B. melitensis dog isolates displaying the specific bands at 731 bp

**Fig. 2:**
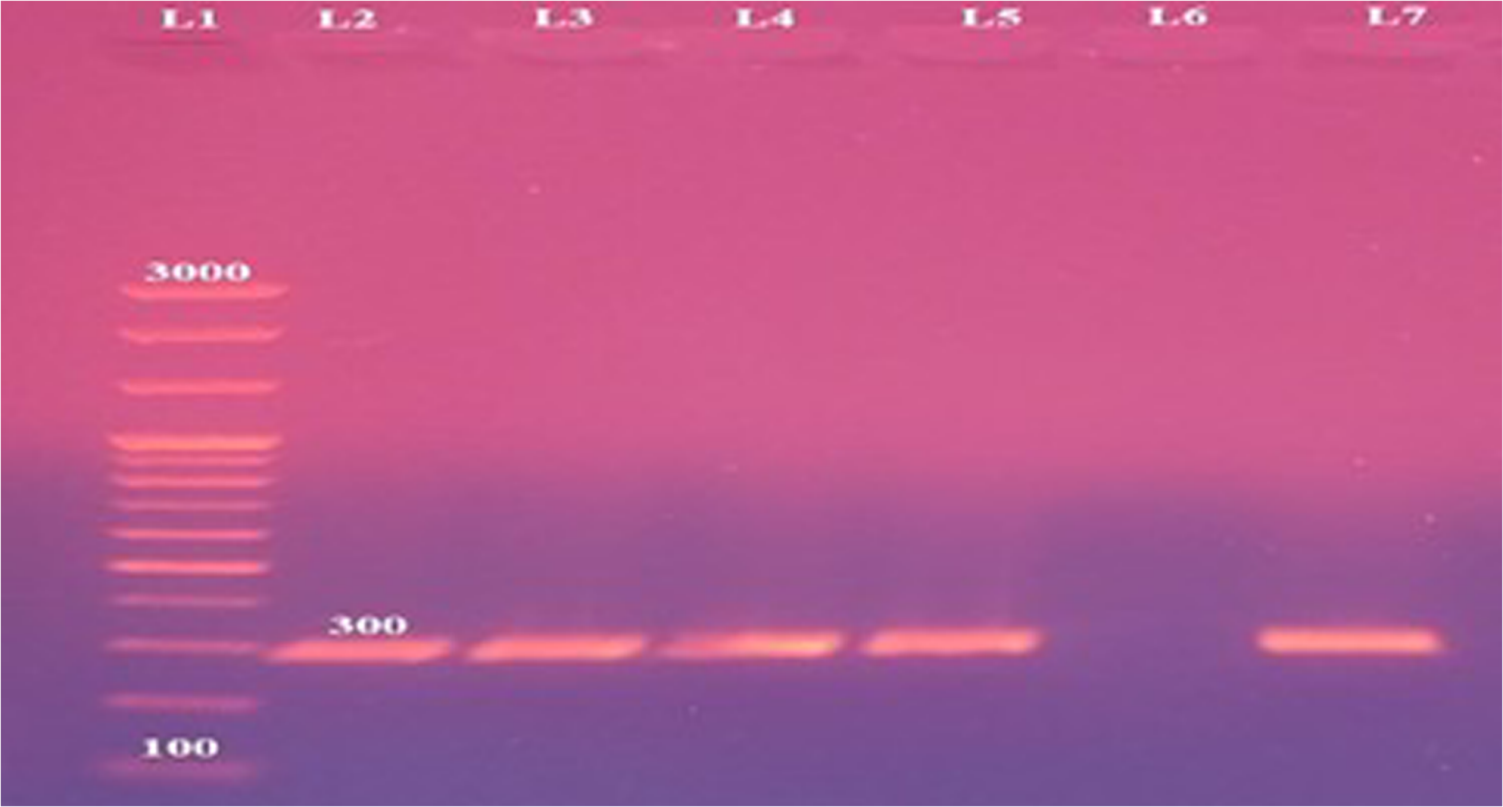
Brucella canis species-specific polymerase chain reaction displaying specific bands at 300 bp for B. canis. Lane 1: 100 bp plus DNA ladder, Lanes 2-5: the 300 bp amplicons specific for B. canis, lane 6: negative control and lane 7: B. canis reference strain RM 666 showing the 300 bp amplicon

AMOS-PCR results showed that 8 out of the 12 isolates (66.66% of brucella isolates; 2.19% of total samples) generated the 731 bp amplicon specifically for *B. melitensis.* These included 3 isolates from uteri of stray bitches, 3 from testicles of stray dogs, and 2 from testicles of owned German Shepherd dogs (Fig. 1). The remaining 4 isolates were negative in AMOS-PCR, indicating they’re not being *B. abortus*, *B. melitensis*, *B. ovis*, or *B. suis*. Species-specific PCR confirmed these isolates as *B. canis*, producing the expected 300 bp amplicon (Fig. 2). All *B. canis* isolates (*n* = 4) originated from stray dogs (2 testicular and 2 uterine samples). Agglutination testing revealed that *B. melitensis* isolates reacted with anti-A and anti-M monospecific sera, whereas *B. canis* isolates reacted only with anti-R monospecific serum. All *B. melitensis* isolates were classified as biovar 3.

### Suis-ladder PCR confirmation

**Fig. 3:**
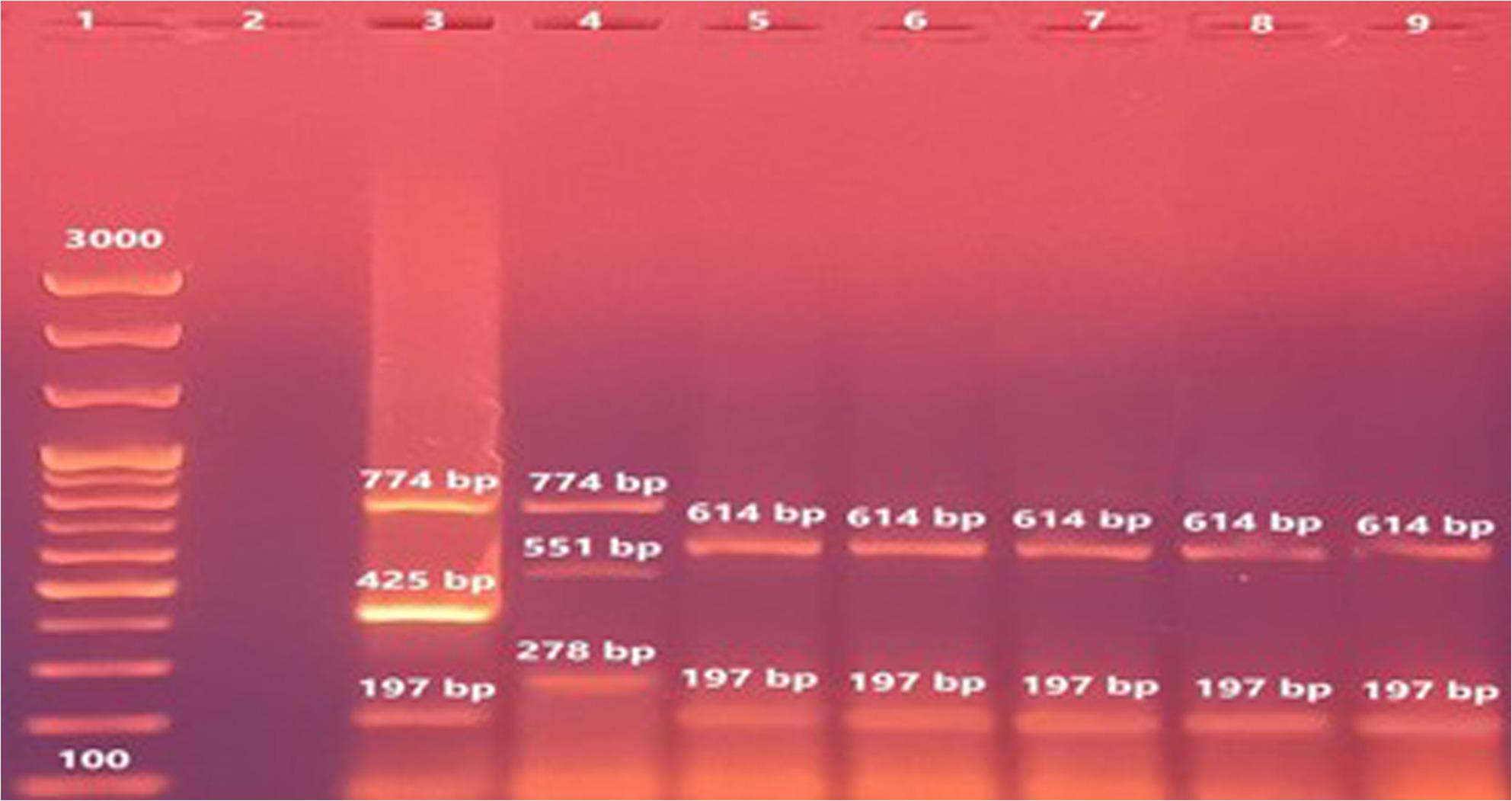
Agarose gel electrophoresis of Suis-ladder multiplex PCR products on DNA isolated from brucella cells. Lane 1: 100 bp DNA size marker, lane 2: Negative control (no DNA template) Lane 3: A reference B. suis biovar 1 strain 1330, lane 4: A reference B. suis biovar 2 strain Thomsen, lane 5: B. canis reference strain (RM666) and lanes 6-9: B. canis isolates recovered in the study

Suis-ladder PCR analysis (Fig. 3) confirmed the identity of *B. canis* isolates through the presence of two characteristic bands (614 and 197 bp). Reference strains of *B. suis* biovars 1 and 2 yielded their respective banding patterns: 774, 425, and 197 bp for biovar 1, and 774, 651, and 278 bp for biovar 2.

### ERIC-PCR phylogenetic analysis

**Fig. 4:**
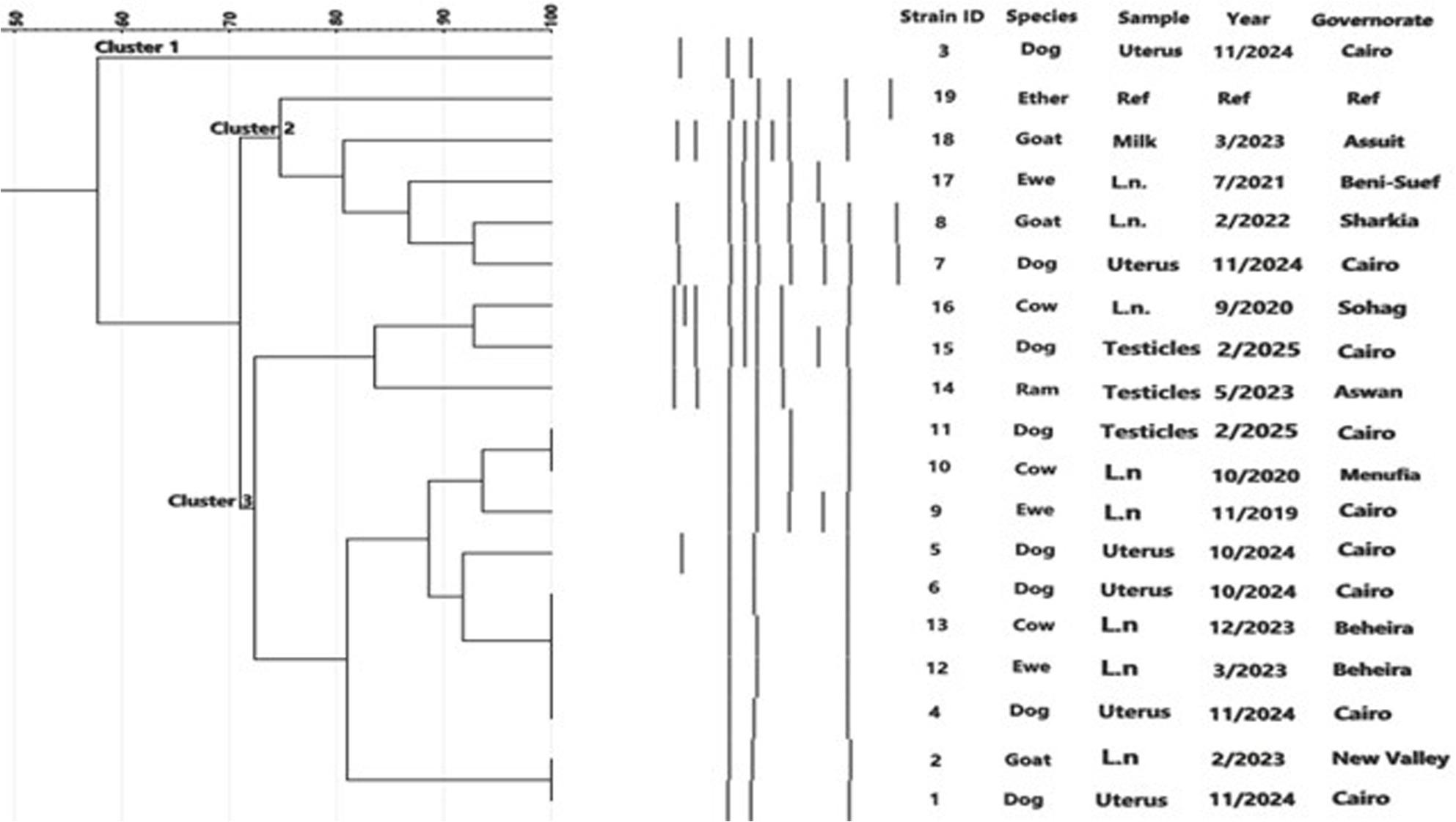
Cluster analysis of ERIC-PCR fingerprints of 18 isolates of B. melitensis recovered in Egypt (8 from dogs in this study) along with the reference strain Ether. Band profiles of each isolate correspond with the lines of the dendrogram. Three major clusters 1, 2 and 3 (similarity ~ 71%) are demarcated. The Brucella spp., animal species, specimens, governorates, years of isolation and strain serial numbers are represented in the columns

**Fig. 5:**
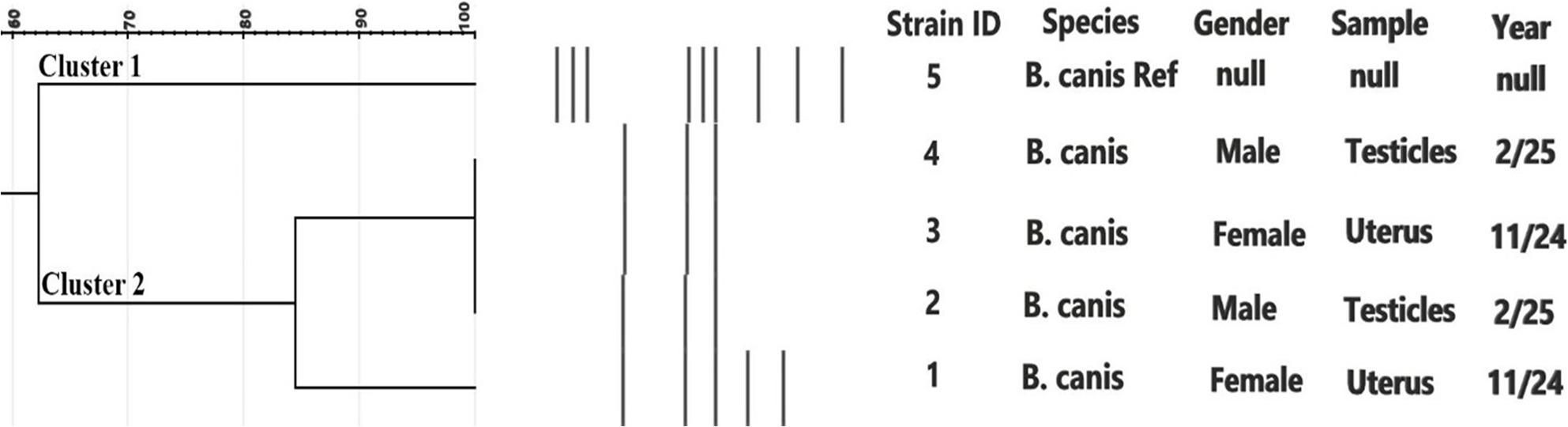
Cluster analysis of ERIC-PCR fingerprints of 4 isolates of B. canis isolated from dogs at Cairo, Egypt along with the reference strain RM/666. Band profiles of each isolate correspond with the lines of the dendrogram. Two major clusters 1 and 2 (similarity ~ 62%) are demarcated. The Brucella spp., specimens, gender, years of isolation and strain serial numbers are represented

All 12 isolates were subjected to ERIC-PCR in two separate analyses, one for *B. melitensis* and the other for *B. canis*. For *B. melitensis*, ERIC-PCR included the 8 isolates from the current study, a reference strain, and 11 additional isolates from other animal hosts. Cluster analysis revealed three major groups with approximately 71% similarity, with distinct profiles reflected in the dendrogram (Fig. 4). For *B. canis*, the 4 isolates were compared with the reference strain RM/666 producing two main clusters with approximately 62% similarity **(**Fig. 5). Metadata for all isolates, including *Brucella spp*, host species, specimen type, governorate, year of isolation, and strain numbers, are presented in the corresponding figures.

### Temporal distribution of *B. melitensis* and *B. canis* isolates from stray and owned dogs across different districts of Cairo, Egypt

During the study period (February–December 2024), a total of 785 dog samples (both stray and owned) as illustrated in Table 3 were examined for *Brucella spp.* detection. Overall, *B. melitensis* biovar 3 was isolated in five sampling groups, while *B. canis* was detected in two groups. The highest prevalence of *B. melitensis* (2.7%) occurred in stray male dogs sampled in Maadi during February, whereas *B. canis* reached a prevalence of 1.5% among stray dogs from Saqqara in June. Mixed infections with *B. canis* and *B. melitensis* were identified in both stray and owned dogs. Positive isolates were obtained from various specimen types. Geographically, positive cases were distributed across multiple Cairo districts (Maadi, Madinet Nasr, Saqqara, Shubra), highlighting the spread but low-level occurrence of *Brucella spp.* in both owned and stray dog populations.

**Table 3:** Temporal distribution of B. melitensis and B. canis isolates from stray and owned dogs across different districts of Cairo, Egypt

Group ID	Sampling date	Samples No. (Gender)	Type of dogs	Specimen*	No. of Positive confirmed Brucella spp	Locality	Prevalence / Month
1	20/2/2024	36 (Males)	Stray	Testicles (*n* = 25) & citrated blood	1 (*B. melitensis bv3)*	Maadi	2.7
2	17/3/2024	58 (Males & Female)	Owned	Citrated blood	-	Misr El Gadida	-
3	10/5/2024	82 (Females)	Stray	Uterus(*n* = 4) & citrated blood	2 (*B. melitensis* bv3)	Madinet Nasr	2.4
4	01/6/2024	195 (Males & Female)	Stray	Uterus(*n* = 59), Testicles(*n* = 67) & citrated blood	3 *B. canis* & *1 B. melitensis*	Saqqara	1.5 (*B. canis*) & 0.5 (*B. melitensis*)
5	26/8/2024	39 (Males & Female)	Owned	Citrated blood	-	Almaza	-
6	28/9/2024	50 (Males & Female)	Owned	Citrated blood	-	Maadi	-
7	29/10/2024	51 (Males & Female)	Owned	Citrated blood	-	Almaza	-
8	12/11/2024	198 (Males & Female)	Stray	Uterus(*n* = 62), Testicles(*n* = 70), & citrated blood	3 *B. melitensis* bv3)	Maadi	1.5
9	5/12/2024	76 (Males & Female)	Owned	Uterus(*n* = 25), Testicles (*n* = 53), & citrated blood	1 *B. canis* & *1 B. melitensis* bv3	Shubra	1.3 (*B. canis*) & 1.3 (*B. melitensis*)

*Blood samples from all examined dogs (*n*=785) were aseptically obtained for serological testing only, however, no Brucella spp. were isolated from any blood specimens

## Discussion

Zoonotic brucellosis caused by *Brucella spp*. remains endemic in several developing countries due to close human–animal interactions [32]. In the present study, *Brucella spp.* was isolated from 12 reproductive tract samples (3.28%), including seven testicles and five uteri. Their presence in dogs heightens the likelihood of the infection in human through direct contact with affected animals or their secretions [33]. Isolation from genital organs supports the concept of localized or focal infection in dogs, particularly in chronically infected animals in which bacteria may persist in reproductive tissues even when circulating antibody levels are low [34, 35] and [36].

In our study, all blood samples tested negative by serology (SAT and 2-ME) and no isolation was recovered from blood cultures. This is consistent with previous reports indicating that bacteremia in dogs infected with *B. canis* is often intermittent or absent, especially in chronic cases. Several authors have also demonstrated that dogs may remain seronegative despite being infected, due to low antibody levels, delayed seroconversion, or localized infection [37–39] and [40].

Importantly, PCR in our study was performed only on cultured isolates to confirm their identity and not on the original clinical samples. Therefore, the observed discrepancy in our results is between serology and bacterial isolation results, rather than between serology and PCR results. The negative serological results observed in culture-positive animals highlight a well-recognized limitation of SAT and 2-ME in diagnosing canine brucellosis. The 2-ME test, although specific for *B. canis*, shows reduced sensitivity in early infection, and may also yield false-negative results in chronic cases due to low circulating antibody titers [25, 37]. Previous studies have also reported false-negative serology associated with slow seroconversion, low bacterial loads, intermittent bacteremia, and inherent limitations in the sensitivity of available serological assays [40].

Our findings underscore that multiple diagnostic tools are necessary, as serology alone may underestimate Brucella infection, especially in chronic or localized disease [41].

Regarding molecular diagnosis, various PCR assays have been adopted for brucella identification, serving also to differentiate species and sometimes biovars. In this study, AMOS-PCR identified eight isolates as *B. melitensis* (Fig. 1) (2.19% of all samples and 66.66% of brucella isolates) and four as *B. canis* (Fig. 2) (1.09% of all samples and 33.33% of isolates) with a higher prevalence among stray dogs compared to owned dogs which lack veterinary maintenance, hygienic measures, and reproductive control which conclusively may act as important source of infection [35] .

Phenotypic characterization and AMOS-PCR indicated that all eight *B. melitensis* isolates belonged to biovar 3 and consistent with *Qureshi et al.* [1], , who greatly recommends their transmission from livestock, particularly small ruminants, which are the primary hosts of this species and given its high pathogenicity to humans. Similarly, *B. canis* is a recognized zoonotic agent, posing risks especially to immuno-compromised individuals and those with occupational exposure such as veterinarians and animal shelter workers.

This study showed the first report of *B. canis* isolation from dogs in Egypt. A previous study [25] reported *B. canis* illness in stray and companion dogs through serological surveys and PCR detection from buffy coat samples but did not perform bacterial isolation. *B. canis* is mostly transmitted amongst dogs, but the detection of both species in the same geographic locations in this study indicates potential for overlapping with different host species [35, 36].

As shown in Fig. 3 in this study, the performed Suis-ladder PCR revealed that the four *B. canis* isolates produced two amplicons of 614 and 197 bp, whereas *B. suis* biovars 1 and 2 reference strains yielded their specific amplicons (774, 425 and 197 bp for biovar 1; 774, 651 and 278 bp for biovar 2). Suis-ladder is a novel multiplex conventional PCR assay that differentiates all *B. suis* biovars and *B. canis*, advancing on the original Bruce-ladder PCR method which sometimes misidentifies *B. canis* as *B. suis*. The Suis-ladder assay used in this study effectively overcame this limitation. These findings highlight the role of dogs as potential sources capable of transmitting not only *B. canis* but also *B. melitensis* to other hosts, including humans [37].

To investigate the genomic relatedness of *B. melitensis* and *B. canis* isolates, all 12 isolates were subjected to ERIC-PCR in two separate amplification sets, one for each species. ERIC-PCR profiles of 8 *B. melitensis* isolates recovered in Egypt, alongside with the reference strain Ether, revealed three main clusters (1, 2, and 3) with approximately 71% similarity (Fig. 4) which showed preliminary clustering patterns, which may show an additional recent common source of infection, probably from livestock reservoirs. The clustering patterns for both species focus on the potential for localized modes of transmission and the sources of infection and reviewing how dogs help *Brucella spp.* maintenance in the environment [38].

In contrast, cluster analysis of ERIC-PCR fingerprints from the four *B. canis* isolates and the reference strain RM/666 demonstrated band profiles consistent with dendrogram clusters, delineating two major clusters (1 and 2) with approximately 62% similarity (Fig. 5). This suggests that the *B. canis* strains in the study area are not genetically identical, theoretically reflecting several sources of infection or continuing free distribution within different dog inhabitants [39].

Although ERIC-PCR was originally developed for enterobacteria, it has been successfully applied to other genera including *Brucella spp.* [40, 41]. Fortunately, ERIC-PCR provides a fast and gainful method for typing *Brucella spp*, particularly in developing countries that cannot afford more expensive genotyping techniques such as Multiple Locus Variable-number Tandem Repeat Analysis (MLVA) or Whole Genome Sequencing-Single Nucleotide Polymorphism (WGS-SNP) analyses [29].

The results in Table 3 concluded that there are temporal and longitudinal spreading of *B. melitensis* biovar 3 and *B. canis*, detected in stray and owned dogs across Cairo, that underscores their potential role as sources for zoonotic brucellosis in which the intermittent temporal pattern of isolation may signify episodic exposure occurrences instead of persistent transmission, thereby underscoring the significance of focused surveillance at high-risk boundaries [42]. Although the overall occurrence was relatively low, the finding of *B. melitensis*, a species recognized for its significant pathogenicity in humans, in stray dogs from many districts suggests continues transmission from the surrounding livestock, likely through common habitats or contaminated animal products [43] which enable the movement of the pathogen between rural or peri-urban livestock populations and urban settings [44].

This study has several limitations that should be considered when interpreting the findings. PCR was applied only to isolates obtained after culture rather than directly to clinical samples and because culture has limited sensitivity for *B. canis*, some infected dogs may not have been detected. Therefore, the present data cannot be used to estimate the true prevalence of canine brucellosis at the population level. Future investigations using direct molecular detection combined with broader sampling strategies are recommended and we plan to perform MLVA or other high-resolution genotyping techniques in future work to allow more robust comparison with global brucella genotypes.

## Conclusion

This study provides the first report of *B. canis* isolation from reproductive organs of stray dogs in Egypt, alongside *B. melitensis* isolates. The findings underscore the dog’s role as potential transmitters of the zoonotic *Brucella spp.*, posing a risk to animals and public health. The application of molecular techniques such as Suis-ladder PCR and ERIC-PCR proved valuable for accurate identification and characterization. Further surveillance and control measures targeting dog populations are recommended to mitigate the zoonotic risk.

## Supplementary Information

Supplementary Material 1.

## Data Availability

All the data generated or analyzed in this study are included in this published article.
