# Supplementary material for: First isolation and molecular confirmation of Brucella canis in dogs from Egypt
Source: BMC Microbiol. 2026 Feb 20;26:165. doi: 10.1186/s12866-026-04787-1 (PMC12930870; doi:10.1186/s12866-026-04787-1)
Supplement: Supplementary file 1 — Supplementary Material 1. [file 12866_2026_4787_MOESM1_ESM.docx]

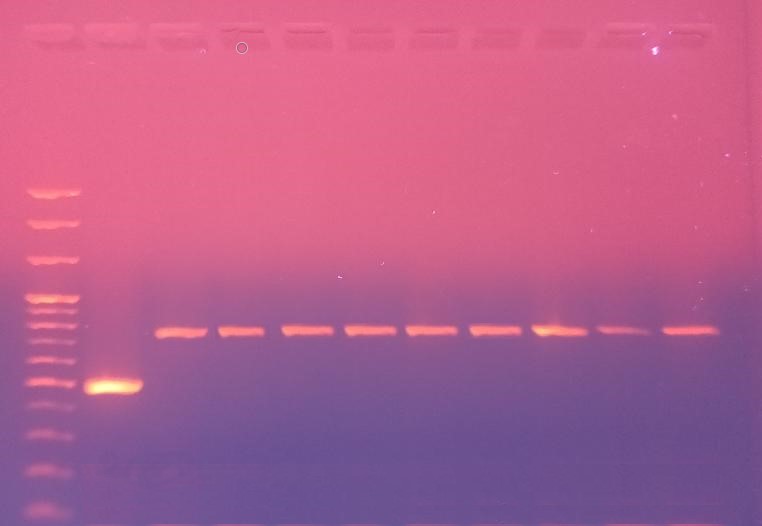


Figure (1): Differentiation of *Brucella species* by AMOS-PCR. Lane 1: 100 bp DNA size marker, lane 2: *B. abortus* reference strain 544; lane 3: *B. melitensis* reference strain Ether and lanes 4-11: *B. melitensis* dog isolates displaying the specific bands at 731 bp.


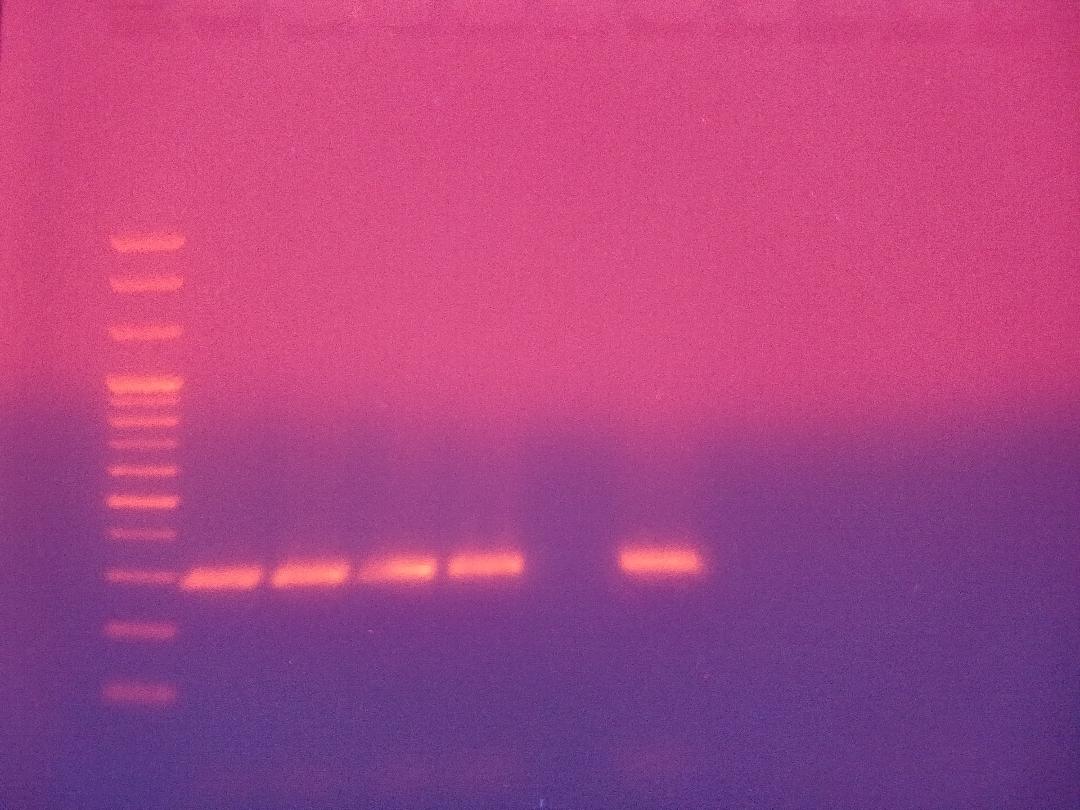


Figure (2): *Brucella canis* species-specific polymerase chain reaction displaying specific bands at 300 bp for *B. canis*. Lane 1: 100 bp plus DNA ladder, Lanes 2-5: the 300 bp amplicons specific for *B. canis*, lane 6: negative control and lane 7: *B. canis* reference strain RM 666 showing the 300 bp amplicon.


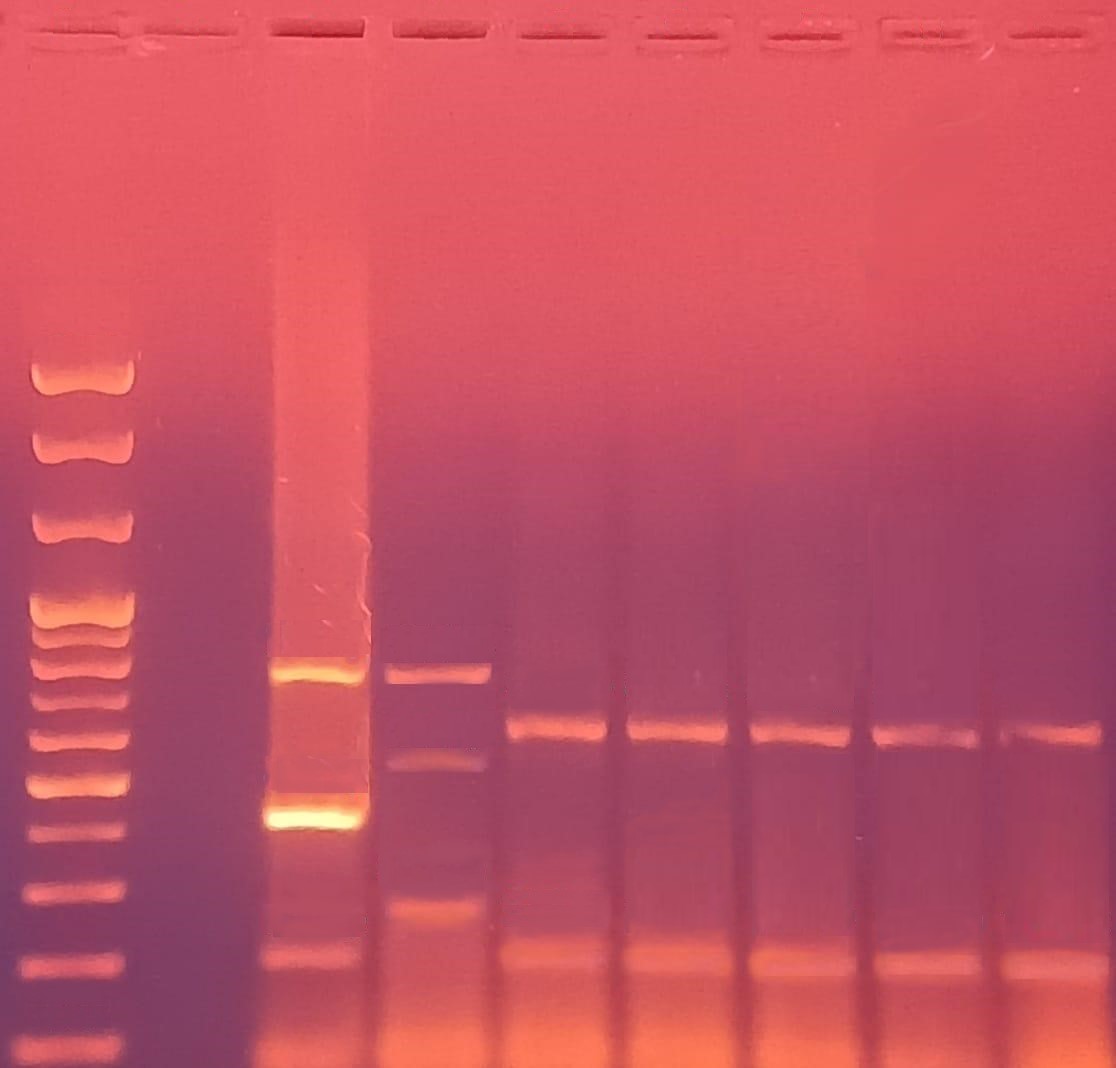


Figure (3): Agarose gel electrophoresis of Suis-ladder multiplex PCR products on DNA isolated from brucella cells. Lane 1: 100 bp DNA size marker, lane 2: Negative control (no DNA template) Lane 3: A reference *B. suis* biovar 1 strain 1330, lane 4: A reference *B. suis* biovar 2 strain Thomsen, lane 5: *B. canis* reference strain (RM666) and lanes 6-9: *B. canis* isolates recovered in the study.
